# Enhancing Zero-Shot Stance Detection with Contrastive and Prompt Learning

**DOI:** 10.3390/e26040325

**Published:** 2024-04-11

**Authors:** Zhenyin Yao, Wenzhong Yang, Fuyuan Wei

**Affiliations:** School of Computer Science and Technology, Xinjiang University, Urumqi 830046, China; 107552103571@stu.xju.edu.cn (Z.Y.); wfv@stu.xju.edu.cn (F.W.)

**Keywords:** zero-shot stance detection, social networking, prompt learning, contrastive learning

## Abstract

In social networks, the occurrence of unexpected events rapidly catalyzes the widespread dissemination and further evolution of network public opinion. The advent of zero-shot stance detection aligns more closely with the characteristics of stance detection in today’s digital age, where the absence of training examples for specific models poses significant challenges. This task necessitates models with robust generalization abilities to discern target-related, transferable stance features within training data. Recent advances in prompt-based learning have showcased notable efficacy in few-shot text classification. Such methods typically employ a uniform prompt pattern across all instances, yet they overlook the intricate relationship between prompts and instances, thereby failing to sufficiently direct the model towards learning task-relevant knowledge and information. This paper argues for the critical need to dynamically enhance the relevance between specific instances and prompts. Thus, we introduce a stance detection model underpinned by a gated multilayer perceptron (gMLP) and a prompt learning strategy, which is tailored for zero-shot stance detection scenarios. Specifically, the gMLP is utilized to capture semantic features of instances, coupled with a control gate mechanism to modulate the influence of the gate on prompt tokens based on the semantic context of each instance, thereby dynamically reinforcing the instance–prompt connection. Moreover, we integrate contrastive learning to empower the model with more discriminative feature representations. Experimental evaluations on the VAST and SEM16 benchmark datasets substantiate our method’s effectiveness, yielding a 1.3% improvement over the JointCL model on the VAST dataset.

## 1. Introduction

Stance detection aims to automatically identify an individual’s stance or attitude (e.g., favor, against, or neutral) expressed in text towards a specific proposition, topic, or target [1,2,3,4]. Traditionally, this task has focused on learning classifiers to predict stances on the same topic. However, in real-world scenarios, with the continuous emergence of new topics, it is impractical to train a classifier for each topic due to the time-consuming and expensive nature of the process. Therefore, zero-shot stance detection, which seeks to recognize stances towards unseen targets based on knowledge of visible targets, has gradually gained attention.

To tackle the issue of zero-shot stance detection, existing research has endeavored to explore attention mechanisms [5], contrastive learning [6], adversarial learning [7], or graph architectures based on external commonsense knowledge [8]. However, these methods often face limitations in capturing capabilities and a dependency on external resources, thereby failing to fully leverage the intrinsic information contained within datasets. On the other hand, with the widespread adoption of pretrained models such as BERT [9] and GPT [10] in the field of natural language processing, prompt learning has emerged as a novel technique, thus demonstrating significant potential in addressing zero-shot stance detection tasks. This approach transforms text classification tasks into cloze tests, where the pretrained language model is responsible for selecting the appropriate word from a set of candidates to fill in the blanks, thereby ensuring semantic alignment with another piece of text. For example, in identifying the sentiment of a social media post like “I missed the bus today”, we might continue with a prompt like “I felt so__” and ask the PLM to choose from a set of emotion-related words to complete the sentence. In this manner, by selecting suitable prompts, we can manipulate the model’s behavior to predict the desired output using the pretrained LM itself, sometimes without the need for any additional task-specific training [11].

Brown et al. [12] first introduced the concept of prompts in the context method. Subsequently, Schick and Schütze [13] proposed PET, which achieves improvements by leveraging patterns in natural language understanding. Some studies [14,15,16] have automated the search for prompts to reduce the dependence on manual pattern design by human experts. All these methods utilize natural language as prompts; hence, they are referred to as discrete prompts. Other methods such as Ppt [17], Prefix-tuning [18], and P-tuningV2 [19] replace natural language prompts with trainable continuous tokens, thereby automatically searching for the optimal prompts in high-dimensional space. Accordingly, these methods are known as continuous prompts. Current prompt-based learning methods typically train models targeting specific task objectives, thus seldom considering the applicability of samples to prompts. Although some recent works [20,21,22] have attempted to generate prompts using contextual information, they often overlook how samples influence prompts, thus focusing instead on how prompts contribute to instances. Such methods usually apply the same prompt pattern across all instances, thereby leading to an inability to fully explore the specific associations between instances and prompts, as well as to guide the model to learn knowledge and information most relevant to the task.

To effectively address the issue of insufficient relevance between instances and prompts in the field of natural language processing, this paper proposes an innovative solution. We introduce a gated mechanism at the core, the gated multilayer perceptron (gMLP) [23], to capture and refine the relevance between instances and prompts precisely. Through this mechanism, we can calculate a relevance score that is used to dynamically adjust the influence of prompts on instances. This not only strengthens the association between instances and prompts but also achieves effective control of the information flow, thereby enhancing the precision and efficiency of the processing.

Furthermore, this study ingeniously integrates strategies from prompt learning and contrastive learning. Prompt learning stimulates the model’s sensitivity to specific tasks by designing appropriate prompts, while contrastive learning enhances the model’s discriminative power by comparing differences between various instances. This combination not only improves the model’s ability to capture subtle differences but also enhances its understanding of complex relationships.

The main contributions of this paper are as follows:

- We propose a novel stance detection model that combines the advantages of prompt learning and contrastive learning, thus enabling effective stance detection in zero-shot scenarios.
- We introduce a gating mechanism that can dynamically adjust the influence of the gate on prompt tokens based on the semantic features of the instance, thereby enhancing the relevance between instances and prompts.
- We conducted experiments on two benchmark datasets, VAST and SEM16, and the results demonstrate that our model outperforms existing state-of-the-art methods on both datasets.

## 2. Related Work

### 2.1. Zero-Shot Stance Detection

Early research on zero-shot stance detection methods largely focused on stance detection within a set of targets, i.e., detection tasks where the training and test sets share the same targets [24]. Crosstarget stance detection is a task similar to zero-shot stance detection, in which a classifier trained on a known target is used to predict stances on data for an unknown target [25]. Existing crosstarget stance detection studies typically utilize models based on attention mechanisms [26] or graph networks [27], thereby learning target-associated features from the training set’s targets and then applying them to predict test sets that are closely related to the target dataset. Unlike crosstarget stance detection tasks, zero/few-shot stance detection aims to automatically determine the stance outcomes for various unknown target data. Under this task requirement, Conforti et al. [28] constructed a large-scale expert-annotated stance detection dataset, where the test set’s targets were invisible relative to the training set. Allaway et al. [5] built a zero-shot stance detection dataset with a wide range of topics, thus covering a broad spectrum of related topic categories. Based on this dataset, Allaway et al. [5] proposed a topic grouping attention model to capture the relationship between targets and general topic representations, but they used a fixed BERT model without further fine-tuning, which significantly limited the model’s performance. In another study, Allway et al. [7] applied a dataset for intratarget stance detection to zero-shot stance detection and employed adversarial learning to extract sample-independent transferable features. However, it required a large amount of unlabeled data from the target, which is not feasible for zero-shot stance detection tasks. Liu et al. [8] introduced relevant commonsense knowledge from both structural and semantic perspectives, thereby proposing a commonsense-enhanced graph model based on BERT to address zero/few-shot stance detection tasks, but they overlooked the relationships between targets. Liang et al. [29] solved this problem using a joint contrastive learning framework and conducting contrastive learning from both context-aware and target-aware perspectives, but their focus was on the contrast between classes, thus ignoring the connections between targets within the same class.

### 2.2. Prompt Learning

Prompt learning is commonly defined as a method that transforms downstream learning tasks into text generation tasks by incorporating prompt information into the text input. Petroni F. et al. [30] introduced the LAMA dataset to test language models’ comprehension of factual and commonsense knowledge. This dataset comprises a set of data sources, each containing a set of facts, which could be in the form of triples or answer pairs. Brown T. et al. [12] created manually crafted prefix prompts for various tasks, including question answering, machine translation, and commonsense reasoning. These prefix prompts demonstrated strong performance across many NLP tasks and benchmarks in zero-shot, one-shot, and few-shot settings. Schick T. et al. [13] targeted text classification and conditional text generation tasks by converting original texts into a “cloze” format using predefined templates, thereby aiding language models in understanding downstream tasks, which is especially challenging in small sample learning settings with only a few samples. Jiang Z. et al. [31], in the MINE method, adopted a mining approach to automatically discover templates from texts containing input x and output y. This method scrapes data from text corpora (like Wikipedia) and then looks for dependency paths between inputs and outputs. Yuan W. et al. [32] used phrase replacements from a thesaurus to translate prompts back and forth between different languages. Wang et al. [33] utilized conceptual knowledge as prompts, thereby enabling models to more effectively understand the nuances of the text and achieving heightened classification accuracy in zero-shot scenarios. Zhu et al. [34] integrated soft knowledge into the prompt tuning process; this strategy markedly improved the model’s grasp of short text contexts, thereby substantially enhancing text classification performance. Goswami et al. [35] introduced a novel lightweight prompt-based method that adapts language models trained on broad domain datasets to various low-resource fields. This method employs domain-specific keywords and trainable gated prompts, thus providing targeted guidance for the intended domain. These studies demonstrate that model guidance through prompt content modification is effective; however, most techniques employ identical prompts across instances, thereby neglecting the specific relationships between instances and prompts. Motivated by these approaches, our study introduces a mechanism using a gated multilayer perceptron (gMLP) to dynamically adjust the impact of prompts on instances, thereby significantly optimizing the model’s performance in stance detection tasks for particular instances.

## 3. Methodology

**Figure 1:**
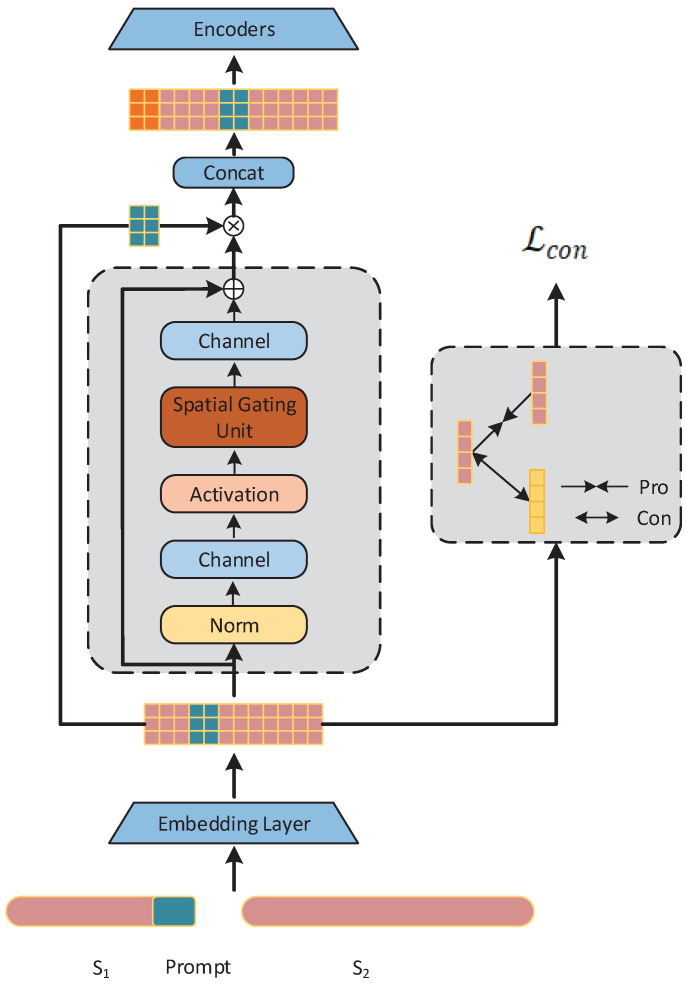
Overall model diagram.

In this section, we introduce the prompt learning method for zero-shot stance detection. We then present the architecture of EZSD-CP, which adds an additional layer between the embedding layer and encoder of pretrained language models (PLMs). This architecture is depicted in Figure 1. The architecture of the EZSD-CP framework mainly consists of six parts: (1) Setting prompt templates to insert prompts between the target and comment text, thus better stimulating the potential of pretrained language. (2) BERT word embeddings, where the target, comment text, and prompt sentences are fed into the BERT model for word embedding to obtain a semantic representation of the text. (3) The gMLP module, which uses gMLP to capture the semantic relevance between instances and prompts and then utilizes this relevance as a gating mechanism to dynamically adjust the influence of prompts on instances. (4) Stance contrastive learning, which performs contrastive learning based on the supervisory signal of stance labels to better generalize stance features and improve the model’s generalization ability. (5) Concat is an integration module that fuses the rich semantic vectors provided by BERT word embeddings with context-sensitive prompt tokens obtained through a carefully designed gating mechanism to generate a comprehensive enhanced feature representation. (6) The encoder module, where we use the deep network architecture of BERT to process word embeddings further, thus obtaining vector representations that include deeper contextual relations.

### 3.1. Task Description

Let M be a pretrained language model (PLM) with a vocabulary V. For a zero-shot stance detection instance ($s_{1}$, $s_{2}$), our goal is to predict the stance of $s_{2}$ towards $s_{1}$, where $s_{1}$ and $s_{2}$ represent the target and comment text, respectively. In prompt learning, $s_{1}$ and $s_{2}$ are typically placed within a specific pattern consisting of special tokens, text pairs, and external prompt tokens. For example, in our task, the instance ($s_{1}$, $s_{2}$) is inserted into a pattern with prompt tokens—[CLS], $p_{1}$, $s_{1}$, [MASK], $p_{2}$, $s_{2}$, [SEP]—and then M is used to select the appropriate word $w\in V^{*}$, where $p_{1},p_{2}\in V_{p}$ are prompt tokens, and $V^{*}$ is the set of candidate label words. Finally, the label word $w\in V^{*}$ is mapped onto the actual labels. In our task, the mapping function is “neutral” → 2, “favor” → 1, and “against” → 0.

(1) $$P\left(y|\left(s_{1},s_{2}\right)\right)=P\left(w|M\left(\left(s_{1},s_{2}\right),p\right)\right)$$

Here, P represents the probability distribution of *y* given the input text pair ($s_{1}$, $s_{2}$), where $p=p_{1},p_{2},$...$,p_{k}$, and *k* is the length of the prompt. Generally, prompt learning is divided into two main categories: discrete and continuous. Discrete prompt learning methods search for human-understandable prompt tokens, meaning that the prompt tokens are a subset of the vocabulary of the pretrained language model (PLM). In contrast, continuous prompt learning methods use pseudo tokens in the pattern, which, during training or inference, are projected into differentiable high-dimensional vectors.

### 3.2. Encoding Module

We use BERT as our pretrained language model and use the coding layer in BERT for word embedding of instances ($s_{1}$, $s_{2}$) and prompts:

(2) $$E=\left[E_{1};E_{p};E_{2}\right]=\mathrm{Embed}\left(\left[s_{1},p,s_{2}\right]\right)$$

where $E\in R^{L_{\times }d}$ is the input embedding matrix, $E_{1}\in R^{L_{1}\times d}$ and $E_{2}\in R^{L_{2}\times d}$ are the embedding matrices of $s_{1}$ and $s_{2}$, respectively, $E_{p}\in R^{k\times d}$ is the embedding matrix of the prompt markers, *L* is the sequence length, $L_{1}$, $L_{2}$, and *k* are the lengths of $s_{1}$, $s_{2}$, and *p*, respectively, and *d* is the dimension of the embedding.

### 3.3. gMLP Module

**Figure 2:**
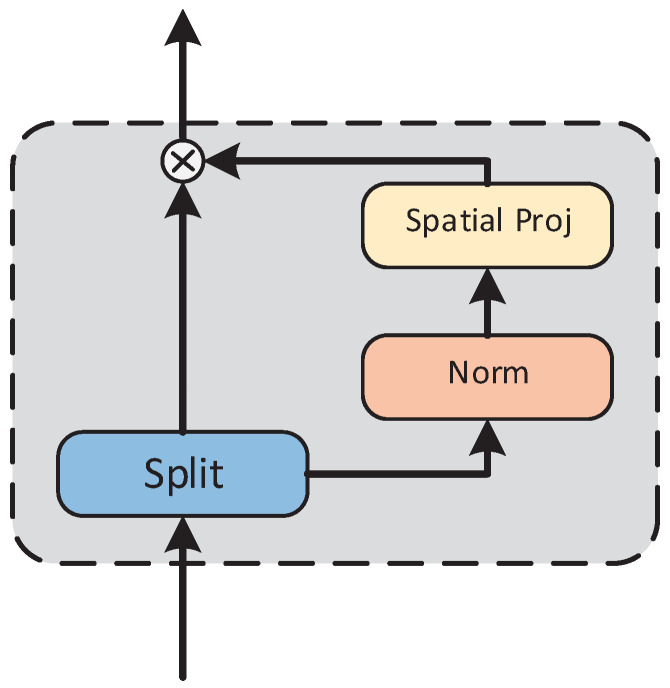
Spatial gating unit.

In the EZSD-CP model, the process of extracting semantic information from instances primarily focuses on effectively extracting information from multiple tokens that compose the prompt. The gMLP model, with its unique structure, such as the spatial gating unit, efficiently processes this semantic information, thereby particularly excelling in understanding and analyzing complex relationships between tokens, as shown in Figure 2. Consequently, we attempt to utilize gMLP to generate channelwise gating signals.

(3) W=σ(Dense(gMLP(E)))

where $W\in R^{k\times d}$ is a weight matrix.

The gMLP consists of a stack of L blocks with the same size and structure. In our model, the input to the gMLP is $E\in R^{L\times d}$. Each block is defined as

(4) $$Z=\sigma \left(EU\right),\;\tilde{Z}=s\left(Z\right),\;Y=\tilde{Z}V$$

where σ is the activation function, and *U* and *V* define linear projections along the channel dimensions—the same as the FFNs of transformers (e.g., they have shapes of 768 × 3072 and 3072 × 768).

One of the key components in the above formulation is s(·), which is a layer capturing spatial interactions, as shown in Figure 2. When *s* is a constant mapping, the above transformation degenerates into a regular feedforward neural network (FFN) in which individual tokens are processed independently without any communication across tokens. For gMLP, a major concern is designing an excellent system that captures complex spatial interactions across tokens. Unlike transformers, the model does not require a positional embedding, as this information will be captured in s(·).

### 3.4. Stance Contrastive Learning

To enhance the generalization ability of stance learning, Gunel et al. [36] proposed a method that defines stance comparison loss on a hidden vector of examples with supervised stance labeling information. The purpose of this loss function is to capture the similarities between examples within the same category and compare them with examples from other categories. Specifically, given a hidden vector ${\left\{hi\right\}}_{i=1}^{N_{b}}$ in a small batch *H* ($N_{b}$ is the size of the small batch), take one of the data $h_{i}$ as an anchor. Among them, $h_{i},h_{j}\in H$. The same label in the same batch is considered a positive pair, i.e., $y_{i}=y_{j}$, where $y_{i}$ and $y_{j}$ are the labels of samples $h_{i}$ and $h_{j}$, respectively. Those with different labels in the same batch are considered negative samples, and then the loss of all positive pairs $\left(h_{i},h_{j}\right)$ and $\left(h_{i},h_{j}\right)$ is calculated as

(5) $$L_{con}=-\frac{1}{N}\sum_{h_{i}\in H}\ell \left(h_{i}\right).$$

(6) $$\ell \left(h_{i}\right)=\log\frac{\sum_{j\in H\setminus i∥[y_{i}=y_{j}]}exp\left(f\left(h_{i},h_{j}\right)/\tau_{s}\right)}{\sum_{j\in H\setminus i}exp\left(f\left(h_{i},h_{j}\right)/\tau_{s}\right)}.$$

where $∥\left[y_{i}=y_{j}\right]\in \left\{0,1\right\}$ is an indicator function; here, its value is 1, $f(h_{i},h_{j})$ is the cosine similarity function for computing $h_{i}$, and $h_{j}$,$f\left(h_{i},h_{j}\right)=\mathrm{sim}\left(u,v\right)=u^{T}v/∥u∥∥v∥$. $\tau_{s}$ is the temperature coefficient for comparison learning.

### 3.5. Concat Module

Finally, EZSD-CP multiplies the prompt embedding and gate weights channelwise and concatenates the new prompt embedding $E_{p}^{\prime }$ with $E_{1}$ and $E_{2}.$

(7) $$E^{\prime }{\;}_{p}=\mathrm{Gate}\left(E_{p};E\right)=W⊙E_{p}$$

(8) $$E^{\prime }=\left[\begin{array}{c} E_{p}^{\prime },E_{1},E_{2} \end{array}\right]$$

where ⊙ stands for channel multiplication, so the continuous prompted learning method in Equation (1) translates to the EZSD-CP:

(9) $$P\left(y|(s_{1},s_{2})\right)=P\left(w|M\left(E^{\prime }\right)\right)$$

### 3.6. Training

The learning objective of our proposed model is to train the model by uniting a supervised stance detection loss $L_{\mathrm{CE}}$ and a contrast learning loss $L_{con}$. The total loss consists of the sum of the two losses:

(10) $$L_{Loss}=\lambda_{c}L_{\mathrm{CE}}\;+\lambda_{n}L_{con}$$

$\lambda_{c}$, $\lambda_{n}$ are tuning hyperparameters, where $L_{\mathrm{CE}}$ is the crossentropy loss. $L_{Loss}$ is calculated as shown in Algorithm 1.
**Algorithm 1** Calculation of the stance contrastive and crossentropy losses
1: **Input:** $s_{1}$,$s_{2}$2: **Output:** 
$L_{Loss}$3: $E\leftarrow \left[E_{1};Ep;E_{2}\right]\leftarrow BERT\_Embedding\left(s_{1},p,s_{2}\right)$▹*p* is the prompt token4:        $L_{con}\leftarrow Contrastive\left(E\right)$▹ Compute contrastive learning loss5: W←σ(Dense(gMLP(E)))▹ Get channel gating6: $E^{\prime }{\;}_{p}\leftarrow W⊙E_{p}$▹ Control prompt weights7: $E^{\prime }\leftarrow \left[E_{p}^{\prime },E_{1},E_{2}\right]$▹ Concatenate processed embeddings with original8: $\hat{y}\leftarrow M\left[E^{\prime }\right]$▹ Classification probability predicted by PLM9:        $L_{CE}\leftarrow CrossEntropy\left(\hat{y},y\right)$▹ Compute classification loss10: $L_{Loss}\leftarrow L_{con}+L_{CE}$▹ Total loss11: **return**
 $L_{Loss}$

## 4. Experiments

### 4.1. Datasets and Evaluation Indicators

Our model was evaluated using the zero/few-shot dataset released in 2020 and the SEM16 dataset published in 2016.

The Varied Stance Topics (VAST) [5] is specifically designed for zero/few-shot stance detection and includes comments from the New York Times “Room for Debate” section, thereby covering a wide range of topics. There are over ten thousand data entries comprising more than 6000 targets. The statistics for VAST are shown in Table 1.

SEM16 contains six predefined targets, including Donald Trump (DT), Hillary Clinton (HC), the feminist movement (FM), the legalization of abortion (LA), atheism (A), and climate change (CC).The statistics for SEM16 are shown in Table 2.

Consistent with previous work, we use the macro average of the F1 scores for each target as the evaluation metric. First, the F1 values for the three categories were calculated, and then the average of the F1 values for all categories was taken.

### 4.2. Experimental Implementation

Our experiments were all encoded using case-insensitive BERTbase with a 12-layer transformer encoder, where each word token was mapped to a 768-dimension embedding. We optimized our model using the Adam optimizer, with all dropout rates set to 0.1. learning rates were chosen from $(1,\;2,\;3,\;4,\;5)\times 10^{-5}$; the training batch size was set to 8, the step size to 0.1, and the final choice of all hyperparameters was based on the performance on the validation set. λc and λn were set to 0.5 and 1, respectively. The learning rates were set to $1\times 10^{-5}$. The median comparative learning loss was chosen from 0.14 to 0.07, both using an A40 graphics card for the experiments.

### 4.3. Baseline Method

To demonstrate viability, we compared the proposed model with the following state-of-the-art models:

- BERT-joint [5]: Contextual conditional encoding followed by a two-layer feedforward neural network.
- TGA Net [5]: The model using contextual conditional encoding and topic-grouped attention.
- BERT-GCN [8]: The model applies the conventional GCN [37] only considering the node information aggregation.
- CKE-Net [8]: A model based on BERT using the CompGCN [38] to obtain the commonsense information.
- DTCL [39]: The model introduces a latent topic cluster embedding and a discrete latent topic variable to build a bridge between various targets.
- ST-PL [40]: The model designs an agent task framework that combines self-supervised learning and cue learning for automatically identifying and exploiting goal-irrelevant gestural expression features while excluding goal-relevant expression features through a data augmentation strategy.
- JointCL [29]: The model consists of stance contrastive learning and target-aware prototypical graph contrastive learning.

### 4.4. Main Results

**Figure 3:**
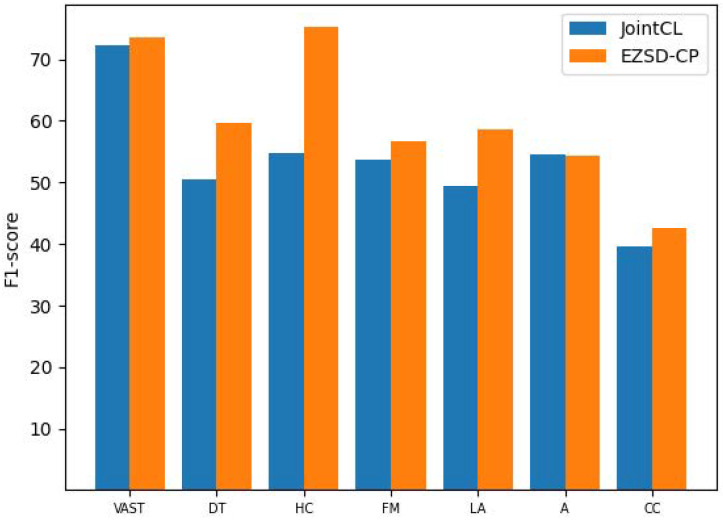
Comparative bar graphs of experimental results of EZSD-CP and JointCL on VAST and SEM16 datasets.

The overall results of our model compared to the baseline are presented in Table 3. To assess the efficacy of our approach across various scenarios, we conducted experiments on the VAST and SEM16 datasets. Our model significantly outperformed all baselines, thereby affirming the effectiveness of our gate mechanism for controlling the influence of prompts on instances and our supervised contrastive learning method. To show our experimental results more clearly, as shown in Figure 3, we compare the experimental results of JointCL and EZSD-CP_bert in a bar chart.

Specifically, our model’s performance on the VAST dataset was two percentage points higher than the ST-PL model. This notable enhancement in performance can be attributed to the combined effect of the adopted gating mechanism strategy and contrastive learning approach. While the ST-PL model, grounded in prompt-based learning, demonstrated commendable capability, our model further refined the dynamic interplay between instances and prompts during the learning process. Moreover, by reinforcing the model’s ability to distinguish features via contrastive learning, we achieved even more impressive results in the challenging zero-shot stance detection task, thereby validating the efficacy of our method.

In the realm of zero-shot stance detection, the JointCL model is regarded as the current best practice due to its enhancement of intercategory connections through clustering. Our model surpassed JointCL in performance, thereby highlighting the significance of introducing gating mechanisms and contrastive learning strategies. The CKE-Net model attempts to strengthen the link between targets and texts by integrating the ConceptNet common sense knowledge graph. In contrast, our model, which capitalizes on the potential of pretrained models through prompt learning, yielded superior outcomes, thus further confirming the effectiveness of prompt-based learning methodologies.

To comprehensively evaluate the generalizability of our model, we experimented with replacing BERT with RoBERTa and compared the results across both datasets. Although the findings on RoBERTa were also encouraging, we noticed a slight decline in performance on the VAST dataset, by 0.6 percentage points compared to BERT. This observation might stem from the multithemed nature of the VAST dataset, where RoBERTa exhibits a finer-grained focus on language comprehension within similar or identical themes. Conversely, on the SEM16 dataset, RoBERTa’s overall performance generally exceeded that of BERT, thus potentially illustrating the inherent advantages of larger-scale pretrained models in zero-shot tasks.

### 4.5. Ablation Experiments

In this study, to delve into the role of each component within the EZSD-CP model, we designed a series of ablation experiments to evaluate the contribution of each component, and the corresponding experimental results are detailed in Table 4. The results of the ablation experiments clearly indicate that once the gMLP gating mechanism was removed, the model suffered significant performance losses across all evaluation metrics. This phenomenon strongly underscores the importance of the gMLP gating mechanism, namely its role in enabling the model to flexibly adjust the response intensity to prompt tokens based on the semantic features of input instances. Furthermore, when we removed the stance contrastive learning (con) component from the model, we observe a significant decrease of nearly six percentage points in the overall performance. This decline reveals the importance of stance contrastive learning within the model, particularly its effectiveness in learning the similarities of stance features within the same category, thereby enhancing the model’s generalization ability to similar targets.

### 4.6. Confounding Experiment

**Figure 4:**
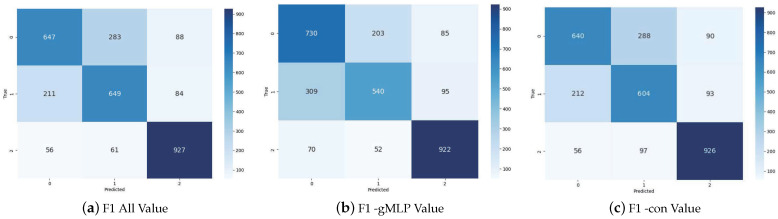
The confusion matrices over different modules. F1 All denotes our proposed model EZSD-CP, F1 -gMLP denotes the removal of the gMLP module, and F1 -con denotes the removal of the contrast learning module.’0’ represents against, ’1’ indicates favor, and ’2’ denotes neutral.

In this experiment, to visually evaluate the model’s performance on the test dataset and accurately reveal the model’s classification efficacy across different categories, we conducted an in-depth confusion matrix analysis for each newly added component of the model. As shown in Figure 4, after the removal of the gMLP module, the recognition accuracy for categories other than the “against” category experienced a decline. This result powerfully indicates the significant role of the gMLP module in enhancing the overall classification accuracy of the model. Furthermore, by combining the data from Figure 4a,c, it can be observed that the prediction accuracy for all categories decreased after the removal of the con module, with the “favor” label experiencing a significant reduction for 40 cases in its prediction accuracy. This change clearly points out the positive contribution of the con module within the model, especially in improving the recognition capability for the “favor” stance. Therefore, these experimental results not only verify the effectiveness of the gMLP and con modules but also highlight their value in constructing an efficient stance detection system.

### 4.7. Analysis and Discussion

**Figure 5:**
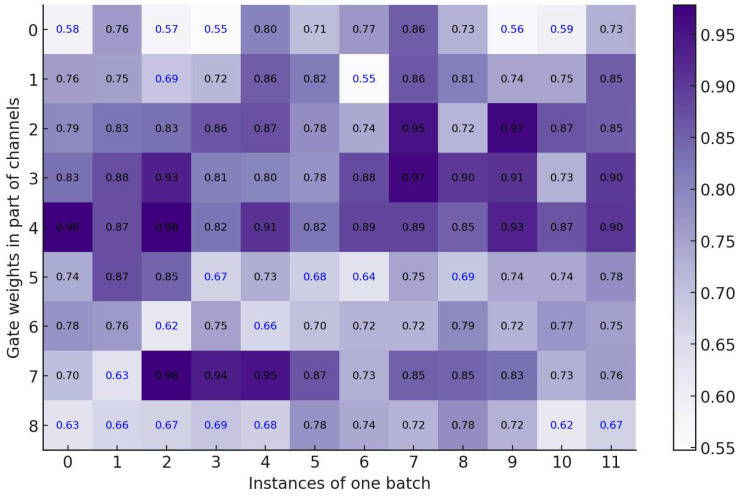
Gate weights in different channels.

We meticulously examined how the gating mechanism influenced the EZSD-CP. We randomly selected 12 instances and analyzed the gating signal weights generated on certain channels for different instances. The visualization results recorded in Figure 5 indicate that the gating mechanism can obtain varying gate weights for different instances.

## 5. Conclusions

In this paper, we elucidate the main problems currently faced by zero-shot stance detection in the context of prompt learning and validate the importance of using a gating mechanism to regulate the influence of prompts on different instances. We proposed an instance-guided prompt learning method, EZSD-CP. EZSD-CP constructs prompts using a weight matrix extracted from instances. Thus, during the training and inference process, the prompts are constrained by the semantic information of the instances. At the same time, contrastive learning was introduced into the model, thereby enabling it to learn more discriminative feature representations. This straightforward approach achieved state-of-the-art performance on both the VAST dataset and the SEM16 dataset.

In the future, we plan to explore a better gating mechanism that can more effectively adjust the influence of prompts on instances based on the semantic information of sentences.

## Figures and Tables

**Table 1:** Detailed statistics of VAST. # denotes “number of” or “count”, indicating the quantity for each category listed.

Statistics	Train	Dev	Test
# Examples	13,477	2062	3006
# Documents	1845	682	786
# Zero-shot Topics	4003	383	600
# Few-shot Topics	638	114	159

**Table 2:** Data statistics for SEM16. DT: Donald Trump, HC: Hillary Clinton, FM: Feminist Movement, LA: Legalization of Abortion, CC: Climate Change is a Real Concern, A: Atheism. # denotes “number of” or “count”, indicating the quantity for each category listed.

Topic	# Ex	# Unlabeled	Keywords
DT	707	2194	trump, Trump
HC	984	1898	hillary, clinton
FM	949	1951	femini
LA	933	1899	aborti
CC	564	1900	climate
A	733	1900	atheism, atheist

**Table 3:** Experimental results on VAST dataset and SEM16 dataset.

Model	VAST (%)				SEM16 (%)					
	Pro	Con	Neu	All	DT	HC	FM	LA	A	CC
BERT-joint [5]	54.6	58.4	85.3	66.1	-	-	-	-	-	-
TGA Net [5]	55.4	58.5	85.8	66.6	41.5	48.7	46.6	45.3	54.2	35.4
BERT-GCN [8]	58.3	60.6	86.9	68.6	42.3	50.0	44.3	44.2	53.6	35.5
CKE-Net [8]	61.2	61.2	88.0	70.2	-	-	-	-	-	-
DTCL [39]	60.0	64.7	87.6	70.8	-	-	-	-	-	-
ST-PL [40]	-	-	-	-	48.4	53.7	51.2	48.1	52.2	35.2
JointCL [29]	64.9	63.2	88.9	72.3	50.5	54.8	53.8	49.5	**54.5**	39.7
EZSD-CP_bert (ours)	**65.4**	**64.5**	**90.6**	**73.6**	59.58	75.2	56.7	58.5	54.48	**42.5**
EZSD-CP_roberta (ours)	65.2	64.3	89.5	73.0	**68.8**	**76.3**	**62.2**	**64.4**	54.44	37.3

**Table 4:** Results of the ablation experiment. The gMLP indicates a gating mechanism, and the con indicates stance contrastive learning.

Model	VAST (%)				SEM16 (%)					
	Pro	Con	Neu	All	DT	HC	FM	LA	A	CC
EZSD-CP (ours)	**65.4**	**64.5**	**90.6**	**73.6**	**59.58**	**75.2**	**56.7**	**58.5**	**54.48**	**42.5**
gMLP	62.1	68.6	85.9	72.2	56.2	74.0	54.9	56.4	35.7	32.0
con	63.6	66.5	88.3	71.6	53.3	73.2	53.4	55.6	37.1	32.9

## Data Availability

The VAST dataset is available at https://github.com/emilyallaway/zero-shot-stance, accessed on 16 August 2023. The SEM16 dataset is available at http://alt.qcri.org/semeval2016/task6/, accessed on 12 September 2023.
